# Indents

**Published:** 1919-04

**Authors:** 


### INDENTS

**☞Brief Articles of Technical or Scientific Value will receive notice and comment. Contributions invited.**

---

DIETETIC VALUE OF NUTS. The war has taught us to appreciate many things which were by no means new, but which had remained in partial oblivion because of the characteristic inertia of man toward a change in his traditional habits. In illustration may be cited the neglect of nuts as articles of foot. Although many persons eat liberally of nuts and the vegetarians have long regarded them as a sort of staple in the diet, we venture to believe that most Americans at least are wont to class them as relishes or accessories. The suggestion to use nuts more liberally often meets the objection that they are not well digested and are likely to give rise to alimentary discomfort. Actual experiment has demonstrated, however, that the charge of inherent indigestibility is not borne out, provided the nuts are consumed under proper conditions. The reported distress attending their use is probably due to inadequate mastication, so that larger masses of an impervious vegetable product are ingested; or they are eaten after an already abundant meal. Nuts are in fact highly concentrated foods, consisting essentially of proteins and fats. Chestnuts alone are rich in carbohydrate, in the form of starch, and are poor in fats. It should be borne in mind, by way of comparison, that the 100-calory portion of most nuts is very small; for example, from twelve to fifteen almonds furnish such an equivalent of food fuel. Remembering that proteins are no longer to be judged solely by their quantity, since a quality factor has been demonstrated by current methods of investigation by direct feeding to be of nutritive significance, likewise it may be asked whether the nut proteins take a reputable rank in this respect. It is probably fair to say that proteins from plant sources do not receive the dietary respect that is accorded to those of animal origin. The proteins of meat, eggs and milk are put into a superior category by a sort of popular consent, when they are contrasted with those furnished by the cereals, legumes and other green vegetables. Some surprise was created, therefore, by Hoobler's experimental evidence of the high value of nut proteins in relation to human milk proteins. A similarly favorable report has just been rendered by Morgan and Heinz from observations they have made on man at the University of California. The protein of almond meal was noted to have a "biologic value" superior to that of wheat gluten, itself a well utilized cereal protein source. Such carefully determined facts must be weighed against popular prejudices in considering the availability of nuts in any regimen. Even though they may not be demanded under the conditions of ordinary diet, there are not a few occasions when carbohydrate-free foods of the protein-fat type would form a welcome addition to the curtailed list that some disease or idiosyncrasy may have occasioned.—*Journal A. M. A.*, March 8.

---

THE POINT OF VIEW. There was a short paragraph in last month's *Dental Record*, garnered no doubt originally for its intrinsic pricelessness, and perhaps selected on this occasion from the editorial scrap-box because it would just fill in a gap on a page? We are not sure. But we do feel sure that when it was inserted the editorial spirit was crowded with lay emotions and the corridors of the editorial mind were irradiated with mirth. The little paragraph is so much too big to fill in a gap on a page. It looks so innocent, like an advertisement or a quotation from a classic and might easily be passed over by a hasty reader. But once read, as its gleaner well knew, it explodes in the mind and flows out over whatever records there may be there of *The Record,* and all other English dental literature, and English dentists and dentistry and beyond into the fastnesses of the consciousness that is shared by English dentists and laymen alike. It is so short, that if only for the benefit of the above-named hasty reader, we may be pardoned for reproducing it. It is from the pages of *The Dental Review.* Here it is: "To me the greatest joy I get out of my practice is making gold foil restorations. If I had to give up gold foil I honestly believe that I would quit the profession. There is no limit to the skill and real art that can be developed in the building of a gold restoration. Here a man can give expression to his very highest ideals. What is more beautiful and artistic than a perfect gold foil restoration? In it you can read the character of the operator as you would an open book." Is there in the United Kingdom a dentist who could produce such a lyric of dentistry, such a whole-hearted, single-eyed enthusiastic *cri du cœur?* We may protest that gold is not in itself æsthetically beautiful, that unrelated blots and squares of gold introduced amidst the rose and ivory of the human mouth are hideously ugly, that the defects in a mouth, the sign and seal of the defects of our civilization could not well be more horribly emphasized than by the glare of polished gold. We may say that there are in the human mouth at least two things that are more beautiful than a perfect gold foil restoration—a perfect natural tooth and a perfect porcelain restoration of an imperfect tooth. We may admit that if gold is more durable, is the most durable filling we can find, it is, whatever its æsthetic defect, worthy of enthusiastic devotion. But can we produce the enthusiasm? Answers come flying from the circumambient English atmosphere. We do not want to. Enthusiasm is not durable. Enthusiasm is not our form. We are an essentially joyous people. Therefore we do not talk, professional Englishmen do not talk, of the joy of their profession. We are a mature individualistic people; not indiscriminately social. America is so socially vitalized that the high pressure of conscious communal life flows into every channel and every American is, in his own right, an enthusiastic specialist. We are differently vitalized, perhaps more from "within" than from "without." Perhaps this is part of the answer to the fact that now that the ideal of a world commonwealth is at last taking material shape, the first nation in the list of "contracting parties" is the United States of America.—*Dental Record.*

---

THE TRIUMPHS OF SURGERY. Under date of December 5th, the *New York Times,* that model of all the virtues and palladium of our public safety, announces that Mr. George J. Gould was operated on at Roosevelt Hospital by Professor Erdman, assisted by Dr. Hayden and another, who removed some gall stones from his kidney. The article further tells something of Mr. Gould's parentage and prospects in life, suggests the size of his income, and makes it apparent that he could afford the remarkable operation that was performed by these ingenious masters of their craft.

From time to time the daily press helps to enlighten the public, hungry for education of all kinds, by little tracts of this sort. We recall one that describes with some detail the removal of a heart, its cleansing and purification and final return to its proper place and function. In the present instance, approaching with all respect the shrine of the city editor, we humbly beseech for light upon the internal arrangements of the exalted sufferer whose gall stones had gotten into his kidney. Perhaps it is like the case of the respectable colored lady, who announced that her case was the most remarkable the Johns-Hopkins Hospital had ever had, because they had cured her of "electric lights of the liver," the interpretation of which proved to be her misconception of Bright's

Disease of the kidney; she gathered that it was something bright and shiny and merely got the physics twisted. One is disposed to wonder upon whose authority Mr. Gould's physiology and anatomy were made public, and whether we will be favored by a monograph from the operating surgeon stamped with the compliments of the author.

The tendency of the daily press to report medical matters for public consumption is something that has irritated the medical profession for years. Possibly no more convincing proof of its futility can be adduced than the columns upon columns published regarding the Spanish influenza and the outbreak of poliomyelitis. As a result of these well meant efforts, the general public was reduced to a state of hysteria, the Health Department received a tremendous amount of publicity, physicians had added burdens placed upon their already overburdened memories and the influenza spread with unchecked speed. The American people and the American press may with propriety take to itself the soliloquy of the bedraggled parrot as he rearranged his torn feathers after a bout with the irritated poodle, whom he had annoyed by his screeching; "Yes," he said, "the trouble with you is you talk too damn much."—Editorial in *Long Island Medical Journal,* by Dr. Webster.

---

Need for More Consultations among Dentists. The wide-awake, thinking, progressive dental practitioner of today is finding himself, suddenly, in a new atmosphere. The ideas and ideals of yesterday are on the scrap heap. A new importance, a wider vision, has come to his profession. He can no longer hedge himself within four walls and live unto himself and by himself. That day is gone, never to return.

The practice of dentistry has assumed such proportions, and the variety of cases presenting at the average office are such, that no one practitioner can be expected to successfully undertake alone and unaided the treatment of every case. If the members of the dental profession have been backward and narrow in any particular, it has been in the matter of calling in a fellow practitioner for consultation over a difficult case about which there may be doubt, or which has not responded to the prescribed course of treatment. But we are at the parting of the ways, and now is the opportune time for our profession to take a saner and broader view of the matter of consultation.

When an important case presents serious difficulties in the matter of diagnosis, or refuses to respond to our course of treatment, we owe it to ourselves and to the best interests of our patient that we state frankly that the case is a troublesome one, is not making the progress that we expected or desired, and that we suggest procuring the advice of another practitioner in consultation. By so doing the intelligent patient will not accuse you of a lack of knowledge or skill, but on the contrary will rightly conclude that your only ambition is to render the best possible service under the circumstances. Also, by following such a course, you have legally and ethically placed yourself in an unassailable position.

How frequently could it be recorded of the dental practitioner, that after a vain effort to cure by the ordinary course of medication an alveolar abscess at the apex of an important tooth, he concludes he is at the end of his resources and informs the patient that nothing further can be done, and advises extraction? That dentist does a great wrong to his patient. His plain duty would be to call to his assistance a fellow practitioner with some knowledge and experience in oral surgery, and if possible, by surgical interference restore to usefulness the diseased organ.

What we are more particularly emphasizing just here, however, is the need for more frequent consultations among the general practitioners in important and stubborn cases. It is true that in the dental profession we are gradually extending our list of specialists, to whom we are more and more referring cases that demand such special knowledge. Yet we must not forget, it is the general practitioner who is the family dentist, and on him falls the burden and responsibility of general mouth conditions, as well as the future health and happiness of his patients. He can not and he should not depend too much on his own individual judgment. Other men frequently have additional knowledge and could make valuable suggestions. We are learning this lesson today as never before. With our post-graduate courses, our society gatherings and our increasing volume of literature, we are mingling socially and professionally to an ever increasing extent. This is to our credit, and to our benefit.

Do not be backward, therefore, in suggesting a consultation: you will be helped, your patient will be helped, and the practice of dentistry will take on new dignity and importance in the eyes of the public.—*Oral Health* for March.

---

Preparation of Dichloramin-T Solution. A five per cent. solution of the salt in chlorinated paraffin, *i. e.*, chlorcosane, answers our purpose quite satisfactorily. We have heard an opinion expressed to the effect that a five per cent. solution is too irritating when used in root canal work. We can not subscribe to such assertions; we rather believe that the pain resulting from its application was due to two causes—a spoiled solution and a faulty technic. Dr. Lee and his co-workers have employed a five per cent. solution in many thousands of cases by pouring quantities of a dram or more directly into an open wound without producing the slightest painful sensation. We can fully confirm these facts.

Solutions of dichloramin-T preserve their activity for a limited time only; they usually deteriorate within two or three months, and therefore it is best to prepare a convenient quantity which may be readily used up within a month or so. To prepare an ounce of the solution, twenty-five grains of dichloramin-T are placed in a dark amber-colored glass-stoppered bottle, which must be absolutely clean and free from moisture. One ounce of chlorcosane is added, the whole is thoroughly shaken, and the bottle is placed in a pan containing very hot water or upon a radiator or other source of indirect heat. Within a quarter of an hour complete solution usually results. Direct heat in making the solution is to be avoided, as it is liable to injure the compound. The solution is immediately ready for use; filtering is not necessary. As stated above, only dark amber-colored or black bottles should be employed as storage vessels; blue glass does not protect the solution against the actinic effects of strong light.

Solutions of dichloramin-T must be carefully protected against heat, light, water, alcohol, and most metals; in fact, most common substances have a strong affinity for chlorin, hence the ready decomposition of this solution when brought in contact therewith. Whenever the solution becomes turbid and forms a deposit of crystals in the bottom of the bottle, or develops a pronounced odor of hypochlorous acid, it should be discarded. Fresh solutions, if chilled, may temporarily become cloudy, or even precipitate, owing to the separation of either dichloramin-T or of solid paraffin. Slightly warming the solution quickly restores its usefulness.

For office purposes it is best to keep the dichloramin-T solution in an amber-colored office-preparation bottle with a ground cap. A small glass rod or tube kept in the bottle readily asists in obtaining the few drops necessary for each treatment, to be placed upon an aseptic glass tray. Under no condition should pliers charged with cotton, etc., be introduced into the preparation in the bottle, and no unused portions of the solution must be returned to the stock bottle.—Herman Prinz, in *Cosmos.*

---

CEMENT DESTROYED BY ALTERATION IN WATER CONTENT.

If a bottle of Caulk's cement liquid is left exposed to the atmosphere, one of two things can happen: it may lose or may absorb water. Under average conditions, the liquid would be more apt to lose water than absorb it. The first effect of this change in the liquid is to upset the balance. Silicate cements are more sensitive to alteration in water content than the zinc oxyphosphates. Evaporation of water from a liquid will cause the cement to become slower setting. For instance, the liquid used in Experiment 1 originally set in less than three minutes, but at the completion of the experiment, that is, after 69 days exposure, of the uncapped bottle, the liquid lost 24.59% of water and required two hours to set. Needless to say, this liquid could not be used in practice, as all dental cement liquids change by exposure.

From time to time, certain dental cement manufacturers have deliberately or ignorantly, advertised their cement liquid as being unaffected by exposure. In most cases, this was intended to enhance the sale of their products by creating the impression that the liquid of other manufacturers, who conscientiously cautioned the user against leaving such liquid exposed, was sensitive, while theirs was unaffected by exposure. The facts in the case are that all dental cement liquids will alter their composition if exposed to the atmosphere. The laws of nature can not be changed to suit the wily advertiser. If a cement is balanced by the manufacturer, this balance can only be preserved by keeping the liquid properly stoppered at all times. A good piece of advice to those who expect excellent cement operation is to keep the liquids properly stoppered at all times, and in as cool a place as possible. If this simple advice is followed, a higher standard in dental operations, involving the use of cement, will result.

